# *Tenebrio molitor* as a Simple and Cheap Preclinical Pharmacokinetic and Toxicity Model

**DOI:** 10.3390/ijms24032296

**Published:** 2023-01-24

**Authors:** Annalaura Brai, Federica Poggialini, Chiara Vagaggini, Claudia Pasqualini, Sauro Simoni, Valeria Francardi, Elena Dreassi

**Affiliations:** 1 Department of Biotechnology, Chemistry and Pharmacy, University of Siena, via A. Moro, 53100 Siena, Italy; 2 Research Centre for Plant Protection and Certification (CREA-DC), via di Lanciola 12/A, 50125 Firenze, Italy

**Keywords:** toxicity assessment, LD50, 3R principles, metabolism, antivirals, *Tenebrio molitor*, animal models

## Abstract

The progression of drugs into clinical phases requires proper toxicity assessment in animals and the correct identification of possible metabolites. Accordingly, different animal models are used to preliminarily evaluate toxicity and biotransformations. Rodents are the most common models used to preliminarily evaluate the safety of drugs; however, their use is subject to ethical consideration and elevated costs, and strictly regulated by national legislations. Herein, we developed a novel, cheap and convenient toxicity model using *Tenebrio molitor* coleoptera (TMC). A panel of 15 drugs—including antivirals and antibacterials—with different therapeutic applications was administered to TMC and the LD50 was determined. The values are comparable with those already determined in mice and rats. In addition, a TMC model was used to determine the presence of the main metabolites and in vivo pharmacokinetics (PK), and results were compared with those available from in vitro assays and the literature. Taken together, our results demonstrate that TMC can be used as a novel and convenient preliminary toxicity model to preliminarily evaluate the safety of experimental compounds and the formation of main metabolites, and to reduce the costs and number of rodents, according to 3R principles.

## 1. Introduction

Toxicity assessment is a fundamental step in drug discovery, because only compounds that demonstrate safety in preclinical trials can progress into clinical phases. Accordingly, several preclinical toxicity models have been developed and used to predict the toxic behavior of new compounds in humans and preliminarily exclude unsafe compounds. Animals differ from humans in many aspects, including body temperature, CYP isoform composition, and expression and catalytic activities of drug-metabolizing enzymes [1]. Each model has strengths and weaknesses based on the information needed; nevertheless, animal models are useful to predict drug toxicity and avoid adverse effects. Mammals are the most used preclinical models, due to their similarity with humans in terms of organs and developmental processes. Because of ethical concerns, preliminary toxicity is usually determined in rodents (mice, rats, rabbits), while efficacy studies are also performed in dogs and non-human primates. The constant demand for novel drugs and better treatments in a limited time, and with relatively reduced costs, is often the cause of failure in the pharmaceutical industry. Several compounds fail due to improper pharmacokinetic (PK) properties or toxicity issues; accordingly, it is fundamental to preliminarily assess PK and toxicity of novel compounds. Since 1959, the “3Rs” concept of replacement, reduction and refinement has been adopted by legislative organs that regulate animal studies [2]. According to 3R principles, the number of animals used in toxicity studies has been reduced, and several in vitro tests have been adopted to preliminarily predict in vivo toxicity. Indeed, alternative non-mammal animal models have been explored as cheap and easier alternatives to acquire preliminary in vivo data. Use of the zebrafish (*Danio rerio*), an Indian fish, to easily study the nervous system in research was pioneered by George Streisinger. Due to its functional similarities with 70% of the disease-causing genes in humans, zebrafish is widely accepted in many disciplines, including developmental genetics, toxicity, cancer and regeneration [3]. *Caenorhabditis elegans* is a nematode that has been widely used in toxicity screening, because the median lethal dose (LD50) in *Caenorhabditis elegans* is comparable with those reported for rats and mice [4]. In recent years, insects have emerged as a potential in vivo model to determine toxicity. Insects are a valuable alternative to preliminarily assess in vivo toxicity and reduce the number of rodents. Indeed, insects are prolific, cheap and can be manipulated by untrained personnel. In addition, their use is less susceptible to ethical concerns and it is not regulated by national legislation. All these aspects are important to reduce the time and costs associated with the first phases of drug development. Several studies demonstrated that insects are reliable models to study the effect of drugs and acquire preliminary information about LD50s. The fruit fly (*Drosophila melanogaster*) represents the first insect used as a model to explore the genetic mechanisms of toxic substances. Recent studies demonstrate the reliability of the fruit fly model in the evaluation of toxicity mechanisms caused by acetaminophen [5]. *Galleria mellonella* is another insect that has been employed since the 1980s as an alternative infection model [6,7,8]. Different studies disclose its use to evaluate the efficacy and toxicity of toxins [9,10], antibacterial [11], antifungal [12,13,14] and antiviral [15,16] compounds. *Tenebrio molitor* larvae (TML) are Coleoptera recently approved for human consumption. Their importance as functional food has been reported in different studies due a composition rich in ACE inhibitory peptides [17,18] and to the possibility of naturally fortifying them using agro-industrial by-products [19,20]. Unlike other insects such as *G. mellonella* or fruit flies, they can be reared in very simple conditions, using different diets and limited spaces, by untrained personnel. The use of TML has been previously reported as an efficacy model for the study of Sporothrix species virulence [21]. With the aim of evaluating in vivo pharmacokinetic (PK) profile and toxicity in reduced times, with low costs and without local authorizations, we analyze herein the toxicity and preliminary PK profile of a panel of drugs in adult exemplars of *T. molitor*. Even if mammalian models remain the “gold standard” of toxicology and, thus, cannot be replaced by invertebrate toxicity models, all these studies demonstrate that TMC can be useful for researchers to gain important information about in vivo toxicity and preliminarily exclude toxic compounds.

## 2. Results and Discussion

### 2.1. Analysis of Vehicles

The list of vehicles used in clinical studies, tolerable levels of nonclinical vehicles disclosed by Gad et al. and available data on *G. mellonella* were taken into consideration [22] to define suitable vehicles for a TMC model. 

*T. molitor* larvae were initially used to perform toxicity studies, adopting the method already developed by Lozoya-Pérez et al., but poor reproducibility was observed in our experiments. In contrast, adult exemplars of *T. molitor* Coleoptera (TMC) proved to be easier to manage and provided us with reproducible results. Considering the limited volume of hemolymph, we considered injecting 1 or 2 µL of vehicles per TMC. The most common vehicles used for i.v. and i.p. studies were administered to experimental groups of six TMC and vitals were monitored daily for seven days. Results are reported in Table 1, expressed as survival %. Physiological saline solution was perfectly tolerated at the volume of 1 or 2 µL. 

DMSO is often used in preclinical tests due to its high solubilizing capability; its toxic dose varies based on the species treated and the route of administration. The reported i.v. LD50 for dogs is 2.5 g/kg; 6.5 g/kg for guinea pigs, 20.6 g/kg for mice, 5.4 g/kg for rats and 100 mg/kg [6] in *G. mellonella*. The administration of 1 µL of DMSO, corresponding to a dose of 8.4 g/kg, was well tolerated; when the dose was increased to 2 µL, a 100% lethality was observed. A 1:1 and a 2:1 mixture of DMSO and saline was administered; even in this case, higher concentrations of DMSO increased mortality. Then, we analyzed other vehicles reported in the literature. Acetone has been well tolerated at the lower dose. When the volume was increased to 2 µL, survival decreased to 16.6%. Reported LD50 for EtOH varies is from 1.6 to 4.3 g/kg in mice and 1.4 g/kg in rats. In our experiments, EtOH was well tolerated at the lower dose of 1 µL (corresponding to a dose of 6.0 g/kg), while increasing the volume to 2 µL led to survival rates decreasing to 50%. 

### 2.2. Toxicity Studies

**Figure 1:**
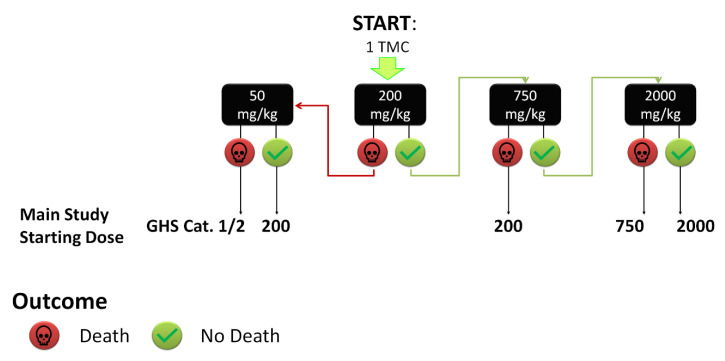
Flowchart for the starting dose selection. When no data were available, we administered a single dose of 200 mg/kg to 1 TMC, increasing or decreasing the dose on the basis of obtained results. GHS cat.: Globally Harmonized System category.

Considering the low solubility of drugs available on the market, 100% DMSO was chosen as a vehicle. The literature was carefully studied to identify the starting dose of a panel of drugs; however, when no data were available, the protocol described in Figure 1 was adopted, using 1 TMC and starting from the dose of 200 mg/kg. 

**Figure 2:**
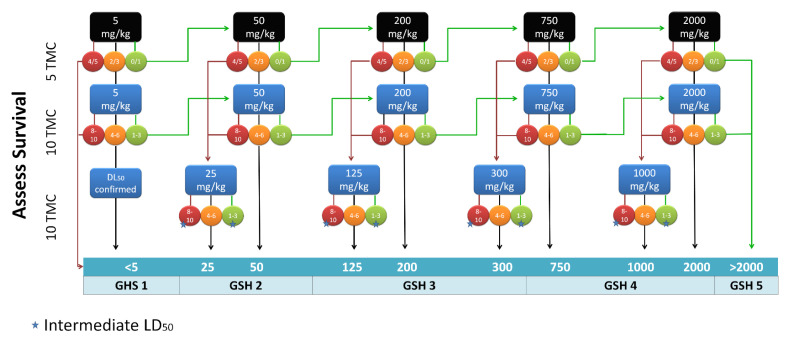
Flowchart for LD50 determination. Starting from the opportune initial dose, LD50 was determined by changing the dose based on results and increasing the number of TML for final determinations. GHS cat.: Globally Harmonized System category. The numbers on the circles represent the number of deaths in exemplars. The star indicates that if the mortality was not 50%, the experiment was repeated using an intermediate dose.

**Figure 3:**
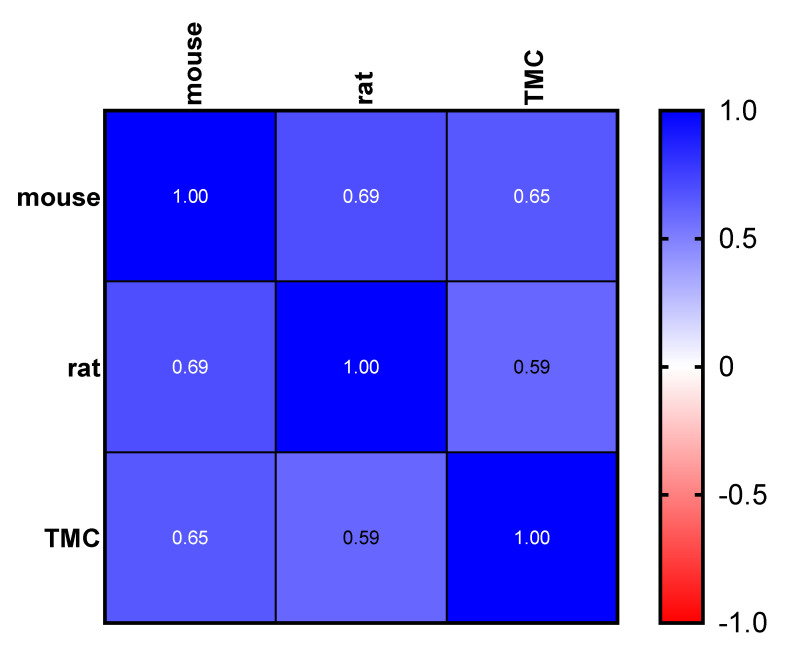
Spearman correlation heat map based on the LD50 dataset obtained from the literature (mice and rats) and experimental LD50 data obtained using TMC model. Spearman r is reported in white. *p*-value < 0.05 was considered significant (TMC vs. rats, *p* = 0.017; TMC vs. mice, *p* = 0.010).

The protocol depicted in Figure 2 has been adopted to determine LD50, starting from the opportune dose determined as described above. A panel of 20 drugs available on the market, including antivirals, antibiotics and the representative drugs for several pharmaceutical classes, was analyzed (Table 2). LD50 data reported in the literature for preclinical tests in mice and rats after intravenous (i.v) and intraperitoneal (i.p.) administrations were compared to obtained data [23,24], showing good correlation (Figure 3). 

An important advantage of the TMC model is represented by the low quantity of materials necessary for the analysis, because TMC body weight is less than that of mice and rats. Acyclovir was not shown to be toxic in our models, because the LD50 was >2000 mg/kg. Higher doses were not evaluated due to the low solubility, in the limited volume of administration, of many compounds. Results are in agreement with Tucker et. al. who reported a dose greater than 650 mg/kg in rats [25]. Amoxicillin possesses an LD50 of 750 mg/kg, lower than that reported for rats after i.p. administration in the Pfizer material safety data sheet (MSDS). Data reported for carbamazepine were comparable to those reported for mice and rats. LD50s disclosed for chlorpromazine (CPZ) and cimetidine were about two times higher than those reported in rodents; on the other hand, the LD50 of cloxacillin was two times lower. The results for codeine, diazepam, diphenhydramine, furosemide, ketoprofen, mepivacaine, metoprolol, salicylic acid, sulfadiazine and trimethoprim were comparable to data reported for rodents. Few data were reported for the recently discovered antiviral molnupiravir after oral administration (LD50 > 800 mg/kg) [26]; in our model LD50 was not determined due to the absence of toxicity at the dose of 2000 mg/kg.

Nimesulide and paracetamol we shown to be more toxic for TMC than for mice and rats [27]. Coman et al. reported significant differences in the LD50 of paracetamol after oral administration [28]. Interestingly, mice and rats have different body temperature regulations and different dose thresholds for paracetamol toxicity. The authors state that the differences are due to the great sensitivity of rats to paracetamol’s ability to cause liver damage (LD50 of 2404 mg/kg with respect to mice’s LD50 of 338 mg/kg). The cause of the higher toxicity for TMC can be due to the absence of temperature control mechanisms. Different studies demonstrate that the external temperature is fundamental for TMC growth and seriously affects the balance between proteins and lipids [29]. High toxicity was also found after treatment with amoxicillin and cloxacillin. Kidneys are the target organs for toxicity of amoxicillin and cloxacillin in rodents and humans; we can postulate that the high toxicity may result from the site of administration being close to the TMC rectal complex and Malpighian tubes. Nevertheless, numerous and poorly understood pathways of systemic toxicity exist, and the mechanisms of toxicity can be different across various systems.

### 2.3. Determination of PK Parameters and Metabolic Stability

Three drugs characterized by high, medium and low in vivo metabolism were selected from our panel of drugs to perform preliminary stability and PK studies [30]. To characterize their phase I metabolism, the drugs were incubated with human or rat microsomes and their main metabolites were determined by HPLC-MS analysis and compared to data already reported (Table 3). 

As indicated in Table 3, furosemide (FUR) was metabolized in both human and rat microsomes; its metabolic stability was about 95%. Its peak area, as well as the presence of its metabolites, were detected on the basis of retention times and *m/z* values. FUR incubation led to the formation of saluamine (CSA), corresponding to the N-dealkylated metabolite, as extensively reported by others [31,32,33]. Carbamazepine (CBZ) stability was about 64 and 77% in rats and humans, respectively; its epoxide (CBZ-E) was detected as the main metabolite, in agreement with studies reported since the 1970s. Chlorpromazine (CPZ) is the subject of extensive metabolism in humans; its in vitro stability was about 87%, and many metabolites were detected by HPLC analysis. The main metabolites detected included desmethyl CPZ (DMCPZ) and di-demethyl DDCPZ, CPZ sulfoxide (CPZSO) and two oxidated compounds [34]. The presence of the same main metabolites of the three drugs were confirmed in TMC, by administrating drugs to three TMC, and analyzing their hemolymph 2 hpt. 

**Figure 4:**
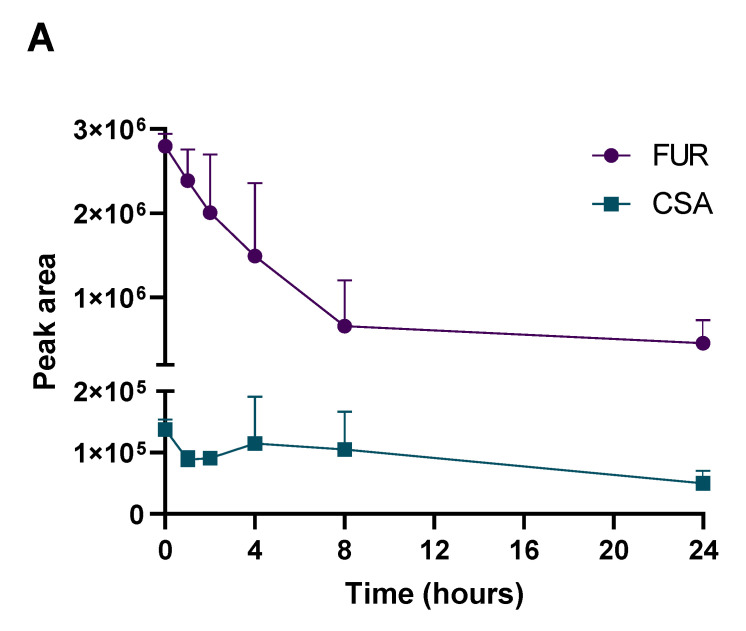

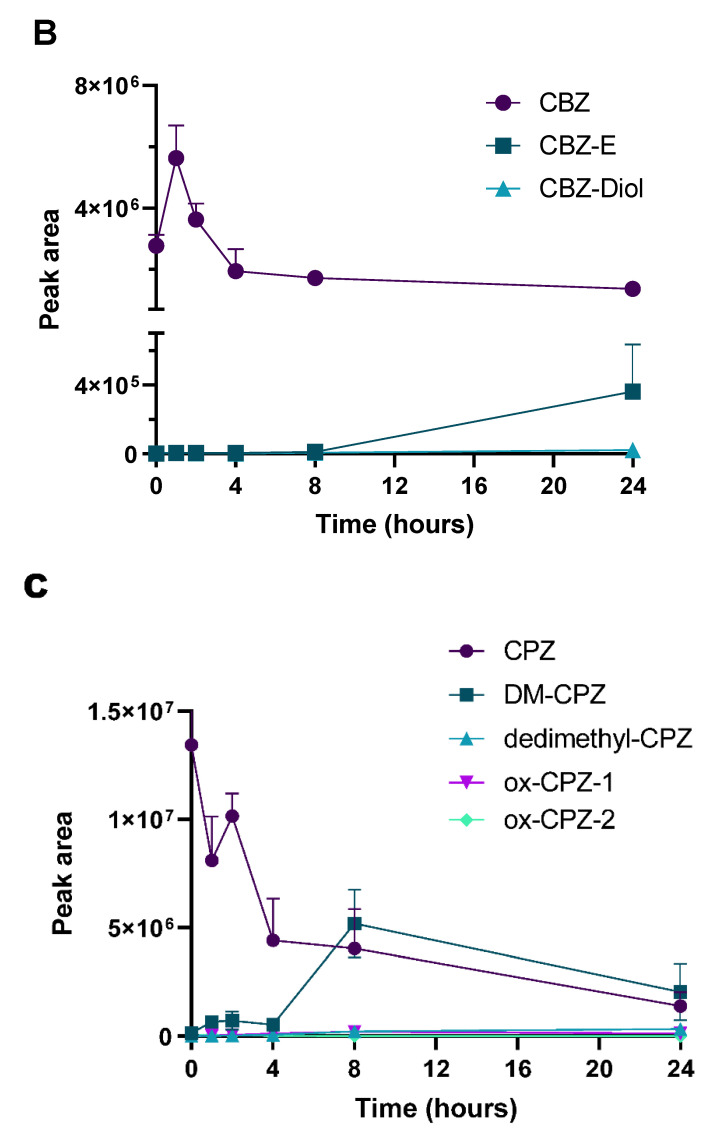
Pharmacokinetic plots of furosemide (FUR) panel (**A**), carbamazepine (CBZ) panel (**B**) and chlorpromazine (CPZ) panel (**C**). The analysis of drugs and the corresponding metabolites was performed at different time points (0, 1, 2, 4, 8 and 24 h post-treatment).

As a preliminary in vivo PK evaluation, FUR, CBZ and CPZ were administered to TMC at the single dose of 62.5 and 150 mg/kg, corresponding to half the LD50, and the areas of the peaks of parent compounds and main metabolites were analyzed. TMC were sacrificed and their hemolymph was analyzed after administration (T_0_) or after 1, 2, 4, 8 and 24 hpt, as described in materials and methods. As shown in Figure 4A, we observed that the elimination of FUR proceeds rapidly. CSA, the most abundant metabolite in humans, was also found in TMC hemolymph, starting from the first time point. CBZ concentration decreased after 4h (Figure 4B), generating a small quantity of CBZ-E, which increased after 8 hpt, reaching the maximum concentration 24 hpt. In addition, CBZ diol was found. As shown in Figure 4C, CPZ was extensively metabolized in TMC, generating a complex mixture of metabolites, in particular, DMCPZ, DDCPZ, CPZSO and other oxidated metabolites. The data were compelling; however, differences among the cytochromes and detoxification mechanisms can be the cause of the differences in PK. *T. molitor* has four major clans of cytochromes (mitochondrial, CYP2, CYP3 and CYP4) implicated in development, detoxification, immune response, digestion, olfaction and reproduction [35]. Their expression varies depending on the organs as well as the reproductive stage and the age of exemplars; as a result, their study complex. Nevertheless, important differences are also present in comparing rats and human microsomes. In our study, FUR, CBZ and CPZ were metabolized more slowly in humans than in rats. In addition, the metabolic capability of young exemplars is higher with respect to elderly people. As an example, the t ½ of CPZ ranges between 8 and 35 h, but can be reduced to 2 h or prolonged to 60 h in some people, depending on age and state of health [36]. All these aspects should be taken into consideration to correctly translate results obtained in more complex models. 

## 3. Materials and Methods

### 3.1. Materials 

All solvents, drugs and reagents used were purchased from Sigma-Aldrich S.r.l. (Milan, Italy) and used without any further purification. 

### 3.2. Animals

TMC used for the experiments were obtained from the mass rearing established at CREA-DC (Florence, Italy), fed on a base diet composed of brewer’s yeast (0.5%) (Laboratorio Dottori Piccioni S.r.l., MI, Italy), wheat flour (49.75%) (Molino F.lli Chiavazza, Casalgrasso, CN, Italy) and oats (49.75%) (Michelotti e Zei, Larciano PT, Italy) [17]. *Pupae* and adult TMC were maintained in semi-dark conditions in a climate room at 27 ± 1 °C and RH (relative humidity) 40–50% [19,20]. Adults that developed from *pupae* were separated and used for analysis within 3 days. 

### 3.3. TMC Injection Procedure

TMC were randomly selected for toxicity assessment. TMC older than 3 days were discarded. Compounds were solubilized in the opportune vehicle and injected on the hemocoel using a Hamilton syringe, (7001 KH, volume 1 µL, needle size 25 s, cone tip), by administering 1 µL of compound on the TMC dorsal side, between the pronotum and elytron. 

### 3.4. Toxicity Testing Procedure

Acute toxicity was determined following the Organization for Economic Cooperation and Development (OECD) guidelines for acute toxicity of chemicals. All the available information on the test substance was considered to select the toxic dose of test compounds. Data were collected considering LD50 already reported for humans, rats and mice or, when possible, insects (e.g., *Galleria mellonella*). The initial dose, expressed in mg/kg, was calculated as the lowest LD50 reported in the literature. When no data were found in the literature, the starting dose of 200 mg/kg was used and the range-finding study was conducted with 1 TMC, by adapting OECD guidelines (Figure 1). To perform LD50 calculation study, 5 TMC were injected with the selected dose, and mortality was recorded daily for 7 days. LD50 was determined by adapting OECD guidelines, as shown in Figure 2. Each experiment included 1 control group treated with DMSO. In detail, if 4 or more TMC died, the compound was re-tested at a lower dose with a new cohort of TMC. If 1 or less TMC died, the experiment was performed with a higher dose. If 2 or 3 TMC died, the experiment was repeated by re-testing the initial dose with 10 TMC. In this case, if 8 or more exemplars died, the experiment was repeated with a lower dose; if 4 to 6 TMC died, the LD50 was confirmed; if 3 or fewer TMC died, the LD50 was considered as a mean of the doses. The experiment continued until a toxic dose was determined. If a compound was not toxic at the highest dose tested (2000 mg/kg), the compound was classified as non-toxic. 

### 3.5. Metabolism Testing Procedure

In total, 6 groups of 3 TMC were treated with carbamazepine, chlorpromazine and furosemide using the procedure described in Section 2.3, using a dose equal to LD50/2. Hemolymph samples were collected after 1, 2, 4, 6, 8 and 24 h post-treatment (hpt). Hemolymph samples were obtained by adapting procedure reported by Tabunoki et al. (2019) [37]. TMC were decapitated and then transferred to the collection tubes. The collection tubes were centrifuged using the 5804 centrifuge (Eppendorf Inc. Co. Ltd. Hamburg, Germany) at 5000 rpm for 10 min, obtaining the hemolymph. 200 µL of ACN was added to each hemolymph sample to denature proteins. The obtained mixture was vortexed and extracted for 10 min with an ultrasound bath. Samples were centrifuged at 5000 rpm for 5 min, and the supernatants were recovered, filtered with a 0.45 µm filter and analyzed using the opportune HPLC method.

### 3.6. In Vitro Metabolism in Human and Rat Microsomes

An amount of 2.5 µL of a solution of the tested compound in DMSO (10 mM) was added to a mixture of phosphate buffer (pH 7.4) and 5 μL of human (HLM) or rat (RLM) liver microsomal protein (0.2 mg/mL) in the presence of an NADPH-generating system at a final volume of 0.5 mL and incubated for 60 min (for HLM) or 30 min (for RLM) at 37 °C. The addition of 1.0 mL of acetonitrile stopped the process. After centrifuging the reaction mixtures, the parent drug and its metabolites were characterized by LC-UV-MS [38]. 

### 3.7. HPLC Analysis

HPLC analysis was performed using an Agilent 1100 LC/MSD VL system (G1946C) (Agilent Technologies, Palo Alto, CA) comprising a vacuum solvent degassing unit, a binary high-pressure gradient pump and a 1100 series UV detector; a 1100 MSD model VL benchtop mass spectrometer was used to perform the chromatographic analysis. Chromatographic separation was obtained using a Phenomenex Kinetex C18-100 Å column (100–4.6 mm, 5 μm particle size). The analysis was performed using a mixture of H_2_O, formic acid 99.9:0.1 (A) and MeOH: ACN: formic acid 49.95: 49.95:0.1 (B). The gradient started with 10% of eluent B, which was linearly increased up to 80% in 10 min, then slowly increased up to 90% in 15 min and, finally, decreased to 10% in 13 min. The injection volume was 20 μL and the flow rate was 0.4 mL min^−1^. The orthogonal spray API-ES was installed on the Agilent 1100 series mass spectra-detection single-quadrupole instrument (Agilent Technologies, Palo Alto, CA, USA). N_2_ was used as a nebulizing and drying gas. The vaporization temperature, capillary voltage, fragmentor voltage, drying gas flow and pressure were set at 40 psi, 9 L/min, 3000 V, 70 V and 350 °C, respectively. UV detection was monitored at 254, 280 and 310 nm. The LC-ESI-MS measurements were performed by operating in either the positive or negative ion mode. Spectra were acquired over the scan range *m/z* 100–1500 using a step size of 0.1 u. Spectra were collected with a 0.1 u step size analyzing the *m/z* 100–1500 scan range. The percentage of non-metabolized analyte was determined by comparison with reference solutions of the testing compounds (carbamazepine (CBZ), chlorpromazine (CPZ) and furosemide (FUR)) solubilized in methanol at the highest concentration administered, 125 or 62.5 mg/kg.

### 3.8. Statistical Analysis

All determinations were run in triplicate, at a minimum, and data were expressed as mean ± standard deviations (SD). All results were subjected to statistical analysis by using one-way ANOVA with a Bonferroni correction post-test using GraphPad Prism, version 5.04 for Windows (GraphPad Software). Spearman’s correlation coefficient was used to analyze the correlation and variability among LD50 obtained in TMC models and data for mice and rat reported in the literature (Figure 3).

## 4. Conclusions

In recent years, insects have emerged as cheap and reliable in vivo models. By eliminating substances with a poor chance of success, our model can easily and inexpensively reduce the gap between in vitro studies and mammalian experiments. Thus, using TMC models can foster drug development programs, reduce the number of mammals in preclinical testing and the overall cost of drug research. Another advantage is represented by the low quantity of compounds necessary for the analysis: about 0.65% and 0.052% compared with mice and rats. Our study demonstrates that TMC may be employed for acute toxicity assessment, yielding results more promptly and affordably than with conventional in vitro and in vivo testing in invertebrates. The fact that the toxicity cannot be tested directly on humans and that it is unknown how experimental LD50 values correlate to human values, or even if the mechanisms of toxicity are the same, is a significant limitation of using insects in toxicity testing, one which also applies to all other toxicity models. The identification of the main metabolites found in rodents and humans suggests that the mechanisms of detoxification and elimination in TMC may be predictive for more complex mammalian models. Numerous and poorly understood pathways of systemic excretion and toxicity exist, and the mechanisms of toxicity can be different across various systems. Even if our model cannot replace testing on mammals before clinical trials, testing in TMC is a useful intermediate step between in vitro tests and testing on mammals, and can be used to preliminarily identify toxic compounds and preliminarily predict PK parameters before more complex mammalian models are used.

## Figures and Tables

**Table 1:** Vehicles analyzed for toxicity studies in adult exemplars of *T. molitor* Coleoptera and survival percentage.

Vehicles	CAS #	Survival %	
		1 µL	2 µL
Saline	7647-14-5	100	100
DMSO	67-68-5	100	0
DMSO: Saline (1:1)	-	100	100
DMSO: Saline (2:1)	-	100	83.4
Acetone	67-64-1	100	16.6
EtOH	64-17-5	100	50

**Table 2:** Drugs analyzed for toxicity studies in adult exemplars of *T. molitor* Coleoptera, LD50 literature data for mice and rats, and experimental data obtained.

Drug	Class	LD50 (mg/kg)				
		Mice		Rats		*Tenebrio molitor*
		*ip*	*iv*	*ip*	*iv*	
Acyclovir	Antivirals	1000	NR	NR	NR	>2000
Amoxicillin	Antibiotics	3590	NR	2870	NR	750
Carbamazepine	Anticonvulsants	114	NR	158	NR	125
Chloramphenicol	Antibiotics		171		NR	300
Chlorpromazine	Neuroleptics	14	106	137	NR	300
Cimetidine	Antiulcer	306	150	328	326	750
Cloxacillin	Antibiotics				1660	750
Codeine	Analgesic	60	54	100	75	50
Diazepam	Neuroleptics	37	25	46.5	32	50
Diphenhydramine	Antihistamines	56	NR	NR	42	50
Furosemide	Loop diuretic		308	800	800	300
Ketoprofen	Anti-inflammatory	300	500	80	350	500
Mepivacaine	Local anesthetic	135	35	NR	30	125
Metoprolol	Antihypertensives	>200	62	NR	71.9	500
Molnupiravir	Antivirals	NR	NR	NR	NR	>2000
Nimesulide	Antipyretic analgesic	NR	NR	300	NR	50
Paracetamol	Antipyretic analgesic	340	NR	NR	NR	125
Salicylic acid	Antipyretic analgesic	300	184	157	NR	250
Sulfadiazine	Antibiotics	750	180	446	880	300
Trimethoprim	Antibiotics	400	132	500	NR	300

NR: not reported.

**Table 3:** PK literature data [30] (a) and in vitro stability data obtained after rat and human liver microsome incubation.

	T ½ (h) ^a^	LogP ^a^	LogS ^a^	Protein Binding (%) ^a^	Vd (L/kg) ^a^	Stability % Rats Microsomes	Stability % Human Microsomes
Furosemide	0.5–2	2.03	−3.66	91–99	0.07–0.2	95.1	96.90
Carbamazepine	5	2.77	−3.20	70–80	0.8–1.9	64.47	77.54
Chlorpromazine	8–35	5.41	−5.01	95–98	7–20	77.99	86.86

## References

[1] Martignoni M., Groothuis G.M.M., de Kanter R. (2006). Species Differences between Mouse, Rat, Dog, Monkey and Human CYP-Mediated Drug Metabolism, Inhibition and Induction. Expert Opin. Drug Metab. Toxicol..

[2] Tsutomu Miki Kurosawa J. (2019). Alternative Research (3Rs) in the World, Asia and Japan. Alternatives to Animal Testing.

[3] Katoch S., Patial V. (2021). Zebrafish: An Emerging Model System to Study Liver Diseases and Related Drug Discovery. J. Appl. Toxicol..

[4] Hunt P.R. (2017). The *C. Elegans* Model in Toxicity Testing. J. Appl. Toxicol..

[5] Saeedi B.J., Hunter-Chang S., Luo L., Li K., Liu K.H., Robinson B.S. (2022). Oxidative Stress Mediates End-Organ Damage in a Novel Model of Acetaminophen-Toxicity in Drosophila. Sci. Rep..

[6] Ignasiak K., Maxwell A. (2017). Galleria Mellonella (Greater Wax Moth) Larvae as a Model for Antibiotic Susceptibility Testing and Acute Toxicity Trials. BMC Res. Notes.

[7] Dinh H., Semenec L., Kumar S.S., Short F.L., Cain A.K. (2021). Microbiology’s next Top Model: Galleria in the Molecular Age. Pathog. Dis..

[8] Desbois A.P., Coote P.J. (2012). Utility of Greater Wax Moth Larva (*Galleria mellonella*) for Evaluating the Toxicity and Efficacy of New Antimicrobial Agents. Adv. Appl. Microbiol..

[9] Champion O.L., Wagley S., Titball R.W. (2016). Galleria Mellonella as a Model Host for Microbiological and Toxin Research. Virulence.

[10] Pérez-Reytor D., García K. (2018). Galleria Mellonella: A Model of Infection to Discern Novel Mechanisms of Pathogenesis of Non-Toxigenic Vibrio Parahaemolyticus Strains. Virulence.

[11] Tsai C.J.Y., Loh J.M.S., Proft T. (2016). Galleria Mellonella Infection Models for the Study of Bacterial Diseases and for Antimicrobial Drug Testing. Virulence.

[12] Li D.D., Deng L., Hu G.H., Zhao L.X., Hu D.D., Jiang Y.Y., Wang Y. (2013). Using Galleria Mellonella-Candida Albicans Infection Model to Evaluate Antifungal Agents. Biol. Pharm. Bull..

[13] Jemel S., Guillot J., Kallel K., Botterel F., Dannaoui E. (2020). Galleria Mellonella for the Evaluation of Antifungal Efficacy against Medically Important Fungi, a Narrative Review. Microorganisms.

[14] Gizińska M., Staniszewska A., Kazek M., Koronkiewicz M., Kuryk Ł., Milner-Krawczyk M., Baran J., Borowiecki P., Staniszewska M. (2020). Antifungal Polybrominated Proxyphylline Derivative Induces Candida Albicans Calcineurin Stress Response in Galleria Mellonella. Bioorg. Med. Chem. Lett..

[15] Antonello R.M., di Bella S., Betts J., la Ragione R., Bressan R., Principe L., Morabito S., Gigliucci F., Tozzoli R., Busetti M. (2021). Zidovudine in Synergistic Combination with Fosfomycin: An in Vitro and in Vivo Evaluation against Multidrug-Resistant Enterobacterales. Int. J. Antimicrob. Agents.

[16] Büyükgüzel E., Büyükgüzel K. (2016). Effects of Antiviral Agent, Acyclovir, on the Biological Fitness of Galleria Mellonella (Lepidoptera: Pyralidae) Adults. J. Econ. Entomol..

[17] Brai A., Immacolata Trivisani C., Vagaggini C., Stella R., Angeletti R., Iovenitti G., Francardi V., Dreassi E. (2022). Proteins from Tenebrio Molitor: An Interesting Functional Ingredient and a Source of ACE Inhibitory Peptides. Food Chem..

[18] Dai C., Ma H., Luo L., Yin X. (2013). Angiotensin I-Converting Enzyme (ACE) Inhibitory Peptide Derived from *Tenebrio molitor* (L.) Larva Protein Hydrolysate. Eur. Food Res. Technol..

[19] Brai A., Vagaggini C., Pasqualini C., Poggialini F., Tarchi F., Francardi V., Dreassi E. (2022). Use of Distillery By-Products as Tenebrio Molitor Mealworm Feed Supplement. J. Insects Food Feed..

[20] Brai A., Poggialini F., Trivisani C.I., Vagaggini C., Tarchi F., Francardi V., Dreassi E. (2022). Efficient Use of Agricultural Waste to Naturally Fortify Tenebrio Molitor Mealworms and Evaluation of Their Nutraceutical Properties. J. Insects Food Feed..

[21] Lozoya-Pérez N.E., García-Carnero L.C., Martínez-Álvarez J.A., Martínez-Duncker I., Mora-Montes H.M. (2021). Tenebrio Molitor as an Alternative Model to Analyze the Sporothrix Species Virulence. Infect. Drug. Resist..

[22] Gad S.C., Spainhour C.B., Shoemake C., Pallman D.R.S., Stricker-Krongrad A., Downing P.A., Seals R.E., Eagle L.A., Polhamus K., Daly J. (2016). Tolerable Levels of Nonclinical Vehicles and Formulations Used in Studies by Multiple Routes in Multiple Species With Notes on Methods to Improve Utility. Int. J. Toxicol..

[23] Williams M. (2013). The Merck Index: An Encyclopedia of Chemicals, Drugs, and Biologicals, 15th Edition Edited by M.J. O’Neil, Royal Society of Chemistry, Cambridge, UK ISBN 9781849736701; 2708 Pages. April 2013, $150 with 1-Year Free Access to The Merck Index Online. Drug Dev. Res..

[24] Wishart D.S., Feunang Y.D., Guo A.C., Lo E.J., Marcu A., Grant J.R., Sajed T., Johnson D., Li C., Sayeeda Z. (2018). DrugBank 5.0: A Major Update to the DrugBank Database for 2018. Nucleic Acids Res..

[25] Techer W.E., Macklim A.W., Szot R.J., Johnston R.E., Elion G.B., de Miranda P., Szczech G.M. (1983). Preclinical Toxicology Studies with Acyclovir: Acute and Subchronic Tests. Fundam. Appl. Toxicol..

[26] Painter W.P., Holman W., Bush J.A., Almazedi F., Malik H., Eraut N.C.J.E., Morin M.J., Szewczyk L.J., Painter G.R. (2021). Human Safety, Tolerability, and Pharmacokinetics of Molnupiravir, a Novel Broad-Spectrum Oral Antiviral Agent with Activity against SARS-CoV-2. Antimicrob. Agents Chemother..

[27] Nelson E.B., Montes M., Goldstein M. (1980). Effectiveness of Methyrapone in the Treatment of Acetaminophen Toxicity in Mice. Toxicology.

[28] Coman L., Păunescu H., Ghiță C.I.V., Țincu R.C., Vasile S., Cinteza D., Fulga I., Coman O.A. (2022). Paracetamol-Induced Hypothermia in Rodents: A Review on Pharmacodynamics. Processes.

[29] Bjørge J.D., Overgaard J., Malte H., Gianotten N., Heckmann L.H. (2018). Role of Temperature on Growth and Metabolic Rate in the Tenebrionid Beetles Alphitobius Diaperinus and Tenebrio Molitor. J. Insect. Physiol..

[30] Ashley C., Dunleavy A., Cunningham J. (2018). The Renal Drug Handbook: The Ultimate Prescribing Guide for Renal Practitioners.

[31] Boles Ponto L.L., Schoenwald R.D. (2012). Furosemide (Frusemide) A Pharmacokinetic/Pharmacodynamic Review (Part I). Clin. Pharmacokinet..

[32] WIRTH P.J., BETTIS C.J., NELSON W.L. (1976). Microsomal Metabolism of Furosemide Evidence for the Nature of the Reactive Intermediate Involved in Covalent Binding. Mol. Pharmacol..

[33] Williams D.P., Antoine D.J., Butler P.J., Jones R., Randle L., Payne A., Howard M., Gardner I., Blagg J., Park B.K. (2007). The Metabolism and Toxicity of Furosemide in the Wistar Rat and CD-1 Mouse: A Chemical and Biochemical Definition of the Toxicophore. J. Pharmacol. Exp. Ther..

[34] Dai J., Lin H., Pan Y., Sun Y., Wang Y., Qiao J.Q., Lian H.Z., Xu C. (2023). xiang Determination of Chlorpromazine and Its Metabolites in Animal-Derived Foods Using QuEChERS-Based Extraction, EMR-Lipid Cleanup, and UHPLC-Q-Orbitrap MS Analysis. Food Chem..

[35] Wang Y.Q., Li G.Y., Li L., Song Q.S., Stanley D., Wei S.J., Zhu J.Y. (2022). Genome-Wide and Expression-Profiling Analyses of the Cytochrome P450 Genes in Tenebrionidea. Arch Insect. Biochem. Physiol..

[36] Yeung P.K.F., Hubbard J.W., Korchinski E.D., Midha K.K. (1993). Pharmacokinetics of Chlorpromazine and Key Metabolites. Eur. J. Clin. Pharmacol..

[37] Tabunoki H., Dittmer N.T., Gorman M.J., Kanost M.R. (2019). Development of a New Method for Collecting Hemolymph and Measuring Phenoloxidase Activity in Tribolium Castaneum. BMC Res. Notes.

[38] Brai A., Riva V., Clementi L., Falsitta L., Zamperini C., Sinigiani V., Festuccia C., Sabetta S., Aiello D., Roselli C. (2021). Targeting DDX3X Helicase Activity with BA103 Shows Promising Therapeutic Effects in Preclinical Glioblastoma Models. Cancers.

